# **Transformation of the Developing World: Socioeconomic Matrix** ^1^

**DOI:** 10.3201/eid1011.040797_03

**Published:** 2004-11

**Authors:** Dennis Carroll, Pierce Gardner, Bradford A. Kay, Michael Osterholm, Edward T. Ryan

**Affiliations:** * U.S. Agency for International Development, Washington, DC, USA;; † National Institutes of Health, Bethesda, Maryland, USA;; ‡ World Health Organization, Lyon, France;; § University of Minnesota, Minneapolis, Minnesota, USA;; ¶ Harvard Medical School, Boston, Massachusetts, USA

Economic disparity affects the health of persons around the world, and various societal, environmental, and economic factors influence the emergence of infectious diseases. Similarly, emerging infectious diseases have a social and economic impact, including diminished economic productivity, increased expenditures on public heath, deferred external investment and development, and reduced travel and retail sales.

The thriving consumer demand for exotic and rare animals as "tonic" food in China, especially in the southern regions, raises concern for the risk for animal-human cross-infections through contact with live and recently slaughtered animals. The increased demand for civet cat, suspected as the source of severe acute respiratory syndrome, is one such example. The demand for tonic food has risen with improving economic conditions in post-1978 China and is a form of conspicuous consumption that expresses economic and social distinction and prestige. A Chinese medical paradigm based on "humors" inherent in the concept of tonic food, combined with the well-understood cultural symbolism of distinction and prestige associated with conspicuous consumption, has lent weight to the demand for rare and exotic animals perceived to be "pure," "safe," and "virile." Since this rising demand is not likely to be suppressible, regulated production of these animals is needed to make them safe.

Additional contemporary issues in China include the effect of migration and urbanization on the spread of sexually transmitted diseases. The forces driving this effect can be divided into three overlapping categories: the dismantling of the organizational and spatial structures that helped keep order in China’s cities during the Maoist era (from 1949 to 1978); a dramatic increase in the overall fluidity of urban societies in China (accompanied by the erosion of traditional moral and behavioral boundaries); and a new set of cultural values that has encouraged more urban Chinese to think of themselves as actors with individual agency. These overlapping forces, which are geographic, socioeconomic, and cultural, are interwoven with and thoroughly implicated in the emergence of new behavior and lifestyles that have put a growing number of Chinese at risk for infectious diseases.

More broadly, climate can also affect public health and emerging infectious diseases. Factors affecting emergence can also be examined in an eco-epidemiologic framework that can often drive epidemics. Examples include the effects of rains and flooding on vector-borne and diarrheal diseases and the effect of heat and fires on respiratory infections.

